# Cannabis oil modulates liver alterations and endocannabinoid system changes in a female rat model of diet-induced MASLD

**DOI:** 10.3389/fnut.2026.1770150

**Published:** 2026-03-10

**Authors:** Valentina Degrave, Michelle Berenice Vega Joubert, Paola Ingaramo, Daniela Sedan, Darío Andrinolo, Tomas M. Mac Loughlin, María Eugenia D’Alessandro, María Eugenia Oliva

**Affiliations:** 1 Laboratorio de Estudio de Enfermedades Metabólicas relacionadas con la Nutrición, Facultad de Bioquímica y Ciencias Biológicas, Universidad Nacional del Litoral, Santa Fe, Argentina; 2 Consejo Nacional de Investigaciones Científicas y Técnicas (CONICET), Santa Fe, Argentina; 3 Instituto de Salud y Ambiente del Litoral (ISAL), Facultad de Bioquímica y Ciencias Biológicas, Consejo Nacional de Investigaciones Científicas y Técnicas (CONICET), Santa Fe, Argentina; 4 Centro de Investigaciones del Medio Ambiente (CIM)/Consejo Nacional de Investigaciones Científicas y Técnicas (CONICET) –Universidad Nacional de La Plata, La Plata, Argentina

**Keywords:** cannabidiol (CBD), cannabis oil, endocannabinod system, MASLD, metabolic syndrome

## Abstract

**Introduction:**

Metabolic dysfunction–associated steatotic liver disease (MASLD) is closely linked to alterations in liver lipid metabolism, oxidative stress, fibrosis, and dysregulation of the endocannabinoid system (ECS). Although increasing evidence supports a role for cannabinoids in metabolic disorders, most preclinical studies have been conducted in male models, leaving female-specific responses largely unexplored.

**Methods:**

This study evaluated the effects of oral administration of a full-spectrum cannabis oil (CBD:THC 2:1) on MASLD-related alterations and ECS regulation in female Wistar rats fed a sucrose-rich diet (SRD). Rats were assigned to reference diet (RD), SRD, or SRD plus cannabis oil (1 mg/kg/day) for 3 weeks.

**Results:**

SRD-fed rats developed liver steatosis and increased NAFLD activity score (NAS), accompanied by enhanced *de novo* lipogenesis, reduced mitochondrial fatty acid oxidation, increased oxidative stress, early fibrotic changes, and ECS overactivation. Cannabis oil administration improved liver lipid metabolism, reduced NAS and fibrosis markers, attenuated lipid peroxidation and oxidative stress, increased NrF2 and decreased NF-κB p65 expression, and normalized hepatic CB1 expression and circulating endocannabinoid levels.

**Discussion:**

These findings demonstrate that full-spectrum cannabis oil is associated with improved MASLD-related outcomes and modulation of ECS tone in a female-specific model of diet-induced metabolic liver disease.

## Introduction

For millions of years, medicinal plants have been employed in the treatment and handling of liver diseases, as well as a range of other health issues such as diabetes, cancer, and malaria. The therapeutic potential of these plants is often linked to their phytochemical and nutritional content (1). One such plant, *Cannabis sativa* L., contains over 600 compounds, including 278 cannabinoids, 174 terpenes, 221 terpenoids, 19 flavonoids, 63 flavonoid glycosides, 46 polyphenols, and 92 steroids (2, 3). Among these, cannabidiol (CBD) and Δ-9-tetrahydrocannabinol (THC) are the primary active compounds that have been the focus of considerable research regarding their interactions. Although many studies have explored the effects of CBD and THC in a variety of disease models, including Metabolic Syndrome (MS), their impact on metabolic dysfunction-associated steatotic liver disease (MASLD) continues to be relatively understudied (4, 5).

Importantly, full-spectrum cannabis preparations contain multiple cannabinoids that may interact to shape their biological effects. The combined administration of CBD and THC has been reported to result in complementary metabolic and anti-inflammatory actions; in particular, CBD may attenuate some of the psychoactive and adverse effects associated with THC while preserving or enhancing its beneficial effects on metabolic and inflammatory pathways (6, 7). The CBD:THC 2:1 ratio was selected based on previous experimental evidence indicating that CBD-enriched combinations retain metabolic benefits while improving tolerability, as well as on preliminary data from our research group showing favorable metabolic outcomes with this ratio in experimental models (8–10). This proportion was therefore considered a biologically relevant and prudent starting point to evaluate the preventive effects of a full-spectrum cannabis oil in the context of diet-induced MASLD.

MS is a group of interconnected metabolic disorders, including central obesity, hypertension, insulin resistance, atherogenic dyslipidemia, oxidative stress, and inflammation, all of these factors notably increase the likelihood of developing cardiovascular disease and type 2 diabetes mellitus. MASLD is commonly recognized as the liver-related aspect of MS, exacerbates the complications associated with the syndrome. While the pathogenesis of MS is complex, it is well-established that a combination of genetic, metabolic, and environmental factors play a crucial role in its rapid progression (11). The global prevalence of MS in adults varies between 12.5 and 31.4%, with differences largely reflecting the definitions used. Higher prevalence rates are observed in middle- and high-income countries, with an increase in frequency linked to age, urbanization, and time. Additionally, MS is more commonly found in women compared to men (12).

The liver, a crucial organ for maintaining and regulating homeostasis, plays an essential role in the detoxification and elimination of both endogenous and exogenous compounds, in addition to being involved in the metabolism of carbohydrates, proteins, and fats. Disruptions in hepatic lipid metabolism can lead to excessive lipid accumulation, resulting in hepatic steatosis. This condition causes oxidative stress within the liver, leading to inflammatory processes. Oxidative stress activates stellate cells and leads to collagen deposition in liver sinusoids, which can progress to necrotic cell death, apoptosis, steatohepatitis, and fibrosis (13, 14).

MASLD has been linked to with dysfunction in the endocannabinoid system (ECS) in both human and animal studies. The ECS is a complex signaling cascade comprising cannabinoid receptors [type 1 cannabinoid receptor (CB1) and type 2 cannabinoid receptor (CB2)], endocannabinoids [such as N-arachidonoyl ethanolamine (AEA) and 2-arachidonoyl glycerol (2-AG)], and various enzymes involved in endocannabinoid synthesis and degradation. Activation of the CB1 promotes the expression of lipogenic genes that are crucial for *de novo* fatty acid synthesis, increases oxidative stress, reduces antioxidant activity, and enhances fibrosis in the liver (15, 16).

Importantly, accumulating evidence indicates that MASLD pathophysiology and ECS function exhibit sex-dependent differences. Sex hormones have been shown to influence hepatic lipid metabolism, oxidative stress responses, inflammatory signaling, and ECS regulation, while cannabinoid pharmacodynamics and metabolism may also differ between males and females. Despite this, most preclinical studies investigating cannabis-based interventions in metabolic disorders have been conducted in male animals, limiting the generalizability of existing findings. This sex bias highlights the need for female-focused experimental models to better understand the biological context in which cannabinoid-based strategies may exert their effects (17, 18).

We have recently demonstrated that non-invasive oral administration of cannabis oil improved arterial hypertension, dyslipidemia, and hepatic steatosis in both male and female Wistar rats fed a sucrose-rich diet (SRD) during 3 weeks (8, 9). In this research, we further investigate the effects in female Wistar rats fed an SRD, with a focus on liver alterations such as lipid metabolism (including *de novo* lipogenesis and β-oxidation of fatty acids), fibrosis, and oxidative stress related to MASLD, as well as changes in the endocannabinoid system (ECS), aiming to identify new molecular mechanisms involved. To our knowledge, no study has explored the impact of a full-spectrum cannabis oil with a CBD:THC 2:1 ratio on MASLD-related parameters and the ECS in a female Wistar rat model. We hypothesize that non-invasive oral administration of cannabis oil (CBD:THC, 2:1 ratio) to female Wistar rats fed an SRD for 3 weeks will improve liver lipid metabolism, reduce NAS scores, prevent oxidative stress and fibrosis, and modulate the tone of the endocannabinoid system (ECS).

## Materials and methods

### Cannabis oil Preparation and Characterization

Cannabis oil was obtained from dried inflorescences of the Cannabis sativa CAT1 variety grown at Environmental Research Center (CIM-CONICET-UNLP) (RESOL-2021–3236-APN-MS). Briefly, in order to obtain neutral cannabinoids, the inflorescences were first decarboxylated in oven (145°C) during 7 min. After that, an alcoholic extraction (10 ml ethanol 96° per gram of inflorescence) was carried out and subsequently the ethanol was evaporated with rotavapor (Buchi R 3000). The resulting resin was diluted in corn oil and the cannabinoids in the oil were quantified by HPLC/UV-DAD techniques. For cannabinoids HPLC/UV-DAD analyses, the extraction was performed with 96° ethanol (Purocol) using 20 mL/g oil and shaking in vortex (10 min) to favor efficient contact and extraction. The obtained solution was then centrifuged for 10 min at 5,000 rpm (Rolco Centrifuge) to separate the alcohol extract from insoluble residues. Finally, the alcoholic phase was filtered using Osmonics 45 lm filters, to further analyze by HPLC/UV-DAD.

Analytical determination of cannabinoids: Cannabinoid profiles were studied by HPLC/UV-DAD (Shimadzu LC-20A), employing a Thermo Hypersil BDS C18 column (150 ⋅ 4.6 mm, 5 lm). The mobile phase consisted in A: methanol and B: 25 mM ammonium acetate solution. The gradient was: 75% A: 1 min, 75–95% A in 15 min, 95% A: 2 min, 95–75% A in 2 min, and 75% A: 5 min. Total run time was 25 min, flow: 1 mL/min, and detection at 205 nm. Cannabinoid analytical standards were purchased from Cerilliant Corporation. The cannabinoid profile of the oil is detailed in Table 1. To prepare the working solution, the oil was diluted to achieve a final concentration of 1 mg/mL.

**TABLE 1:** Quantification of cannabinoids in cannabis oil.

Cannabinoids	Initials	Concentration (mg/mL)
Cannabidivarinic acid	CBDVA	ND
Cannabidivarin	CBDV	ND
Cannabidiolic acid	CBDA	0.34
Cannabigerolic acid	CBGA	0.02
Cannabigerol	CBG	0.02
Cannabidiol	CBD	0.68
Tetrahydrocannabivarin	THCV	ND
Tetrahydrocannabivarinic acid	THCVA	ND
Cannabinol	CBN	ND
Δ-9-Tetrahydrocannabinol	Δ-9-THC	0.47
Δ-8-Tetrahydrocannabinol	Δ-8-THC	ND
Cannabichromene	CBC	0.05
Tetrahydrocannabinolic acid	THCAg	0.03
Total Cannabidiol (CBD + CBDA × 0.877)		0.99
Total Tetrahydrocannabinol (THC + THCA × 0.877)		0.50
CBD:THC ratio		2:1

ND, Not detected.

Peak profile and terpene identification were performed by GC-FID analysis (Agilent Technologies 689ON) Giese et al. (19). Compounds were identified by comparing the retention times with standards (Restek Co.^®^) (Table 2).

**TABLE 2:** Quali-quantitative terpene profiles in cannabis oil.

Terpenes	Concentration (ppm)
Alpha-pinene	ND
Camphene	ND
(-)-beta-pinene	ND
Beta-myrcene	ND
Delta-3-carene	ND
Alpha-terpinene	ND
p-cymene	ND
d-limonene	ND
Cis-b-ocimene	ND
Trans-b-ocimene	ND
Gamma-terpinene	ND
Terpinolene	ND
Linalool	ND
(-)-isopulegol	ND
Geraniol	ND
Beta-caryophyllene	24.1
Alpha-humulene	13.8
Cis-nerolidol	ND
trans-nerolidol	ND
(-)-guaiol	29.8
(-)-alpha-bisabolol	41.1
Total terpenes	108.8

ND, Not detected.

### Animals and diets

Eighteen female Wistar rats were sourced from the Veterinary Sciences Institute of Litoral (ICIVET-Litoral) at the Faculty of Veterinary Sciences, National University of Litoral (Esperanza, Santa Fe, Argentina). The rats were kept under standard conditions with unrestricted access to food and water, regulated temperature (22 ± 1°C), humidity, ventilation, and a light/dark cycle lasting 12 h (lights on from 7:00 a.m. to 7:00 p.m.). Every effort was taken to minimize animal distress, and the number of subjects was kept to a minimum. The research adhered to the NIH standards regarding the treatment and utilization of experimental animals and received authorization from the Institutional Ethics Committee at the Faculty of Biochemistry and Biological Sciences (UNL, Santa Fe, Argentina—Acta 03/21).

Initially, all animals received a standard commercial rodent diet in powdered form (GEPSA FEED, Buenos Aires, Argentina). Animals weighing 130–140 g were randomly assigned to three experimental groups for 3 weeks: (1) a control group that continued on the standard powdered diet (reference diet, RD, *n* = 6), (2) a group given a semisynthetic sucrose-rich diet (SRD, *n* = 6), and (3) a group that received SRD supplemented with orally administered cannabis oil (SRD + Ca, *n* = 6). The cannabis oil used had a CBD:THC ratio of 2:1 or alternatively a vehicle control consisting of corn oil for both RD and SRD groups. The administration occurred daily via an oral syringe at a dosage of 1 mg/kg body weight throughout the 3-week experimental timeline. This specific dosage was chosen based on prior studies by our group (8, 9), which indicated positive effects on metabolic parameters such as arterial hypertension, dyslipidemia, and hepatic steatosis in Wistar rats on an-SRD. Consistent dosing allowed for comparison with earlier research results while further characterizing underlying mechanisms.

The non-invasive oral delivery method employed is favored in preclinical studies due to its speediness, accuracy in dosing administration, ability to reduce stress levels in animals, and potential enhancement of animal welfare (20–22). Detailed compositions of diets can be found in Degrave et al. (8). The rats initiated and concluded the experimental protocol during the same phase of their estrous cycle, specifically either diestrus or proestrus.

Animals were deeply anesthetized with sodium pentobarbital (60 mg/kg, intraperitoneal injection). Adequate depth of anesthesia was confirmed by the absence of pedal withdrawal and corneal reflexes. Euthanasia was performed by decapitation under deep anesthesia. Death was confirmed by the absence of heartbeat and respiratory movements prior to tissue excision. Blood samples were collected from the inferior vena cava, immediately subjected to centrifugation, and the serum obtained was either analyzed right away or stored at –20°C for future use. Each rat’s liver was completely removed, weighed, and sectioned for further analysis. Liver tissue was preserved in 10% buffered formalin for 24 h at room temperature before being embedded in paraffin for histological assessment; other sections were frozen and maintained in liquid nitrogen.

### Analytical methods

The serum concentrations of the endocannabinoids 2-AG and AEA were measured using a previously established technique (8). Lipid peroxidation was assessed through the measurement of substances that react with thiobarbituric acid (TBARS) in both serum and liver, adhering to the protocol specified by Degrave et al. (8). The levels of reactive oxygen species (ROS) in the liver were evaluated according to the method outlined in Ferreira et al. (23).

### Liver histology

Liver tissue sections (5 μm) were stained with H&E, visualized under a bright-field microscope, and analyzed with Image Pro-Plus software to assess steatosis and calculate the NAFLD activity score (NAS) following Vega Joubert et al. (24).

### Enzymatic activity assays

The activities of various enzymes including liver acetyl-CoA carboxylase (ACC), fatty acid synthase (FAS), glucose-6-phosphate dehydrogenase (G-6-PDH), and malic enzyme (ME) were analyzed based on protocols outlined in Degrave et al. (8). Furthermore, the activities of carnitine palmitoyltransferase-1 (CPT-1), CPT-2, and total CPT in liver tissues were evaluated utilizing the methods detailed in Ferreira et al. (23).

### Antioxidant defense system

The evaluation of the non-enzymatic antioxidant glutathione (GSH), along with the enzymatic activities of catalase (CAT), glutathione reductase (GR), and glutathione peroxidase (GPx) in liver tissue, was conducted following established methodologies (8)

### Liver fibrosis

Collagen arrangement within the liver was examined utilizing an Olympus BH2 light microscope (Olympus Optical Co., Ltd., Japan), as previously detailed by Oliva et al. (25). Furthermore, collagen birefringence in liver sections stained with picrosirius red was assessed using polarization microscopy (26, 27). The hydroxyproline content in the liver was measured according to Neuman and Logan’s method from 1950, with minor modifications outlined earlier (28).

### Immunohistochemical analysis

A standard immunohistochemical technique, following protocols previously described (29, 30), was performed. Paraffin-embedded (5 μm thickness) liver cross-sections were used to evaluate the protein expression of 4-HNE, NrF2, NF-κB p65 and TGF-β1. The sections were mounted on 3-aminopropyltriethoxysilane (Sigma-Aldrich, Buenos Aires, Argentine)-coated slides and a subsequent microwave pretreatment for antigen retrieval was performed. The samples were incubated in a humid chamber first with a specific primary antibody for 4-HNE (mouse monoclonal antibody; Catalog # MAB3249; R&D Systems), NrF2 (mouse monoclonal antibody; sc-365949; Santa Cruz Biotechnology), NF-κB p65 (mouse monoclonal antibody; sc-8008; Santa Cruz Biotechnology) and TGF-β1 (mouse monoclonal antibody; sc-52893; Santa Cruz Biotechnology) (for 14–16 h at 4°C) and then with biotin-conjugated secondary antibody (anti-mouse, 1:100 dilution, Sigma) for 30 min at room temperature. The reactions were developed using the streptavidin–biotin peroxidase method and diaminobenzidine (Sigma) as a chromogenic substrate. Each immune-histochemical run included positive consisting of tissue samples with previously confirmed expression of the target antigen, as reported in our prior studies (8, 25), and negative controls obtained by omission of the primary antibody, which consistently resulted in the absence of specific immunostaining. Immunohistochemical analyses were performed using 6 independent biological samples per experimental group, with all sections processed under identical experimental conditions. The expression of 4-HNE, NrF2, NF-κB p65 and TGF-β1 was evaluated by image analysis using the Image Pro-Plus 5.0.2.9 system (Media Cybernetics, Silver Spring, MD, United States). Immunostained images were captured with a Dplan 40 × objective (numerical aperture, 0.65; Olympus) attached to a Spot Insight V3.5 color video camera. Quantification was performed on at least 10 randomly selected fields per section. After convert each image into a gray scale, the integrated optical density (IOD) was measured as a linear combination of the average gray intensity and the relative area occupied by positive cells as was previously described by Ingaramo et al. (31).

### Western blot analysis

Frozen liver samples were homogenized in RIPA buffer supplemented with protease inhibitors, left on ice for 10 min, and the supernatant was collected by centrifugation at 25.000 × g for 10 min, as described by Li et al. (32). Proteins were separated by SDS-PAGE in a 10% gel and electrotransferred onto PVDF membranes. The membranes were probed with mouse primary monoclonal antibodies against CB1 (mouse monoclonal antibody; sc-293419; Santa Cruz Biotecnology) and then incubated with goat anti-mouse IgG conjugated to horseradish peroxidase antibody (mIgG-Fc-BP-HRP; sc-525409, Santa Cruz Biotechnology). Specific signals were visualized using a chemiluminescent detection system (Bio-Lumina, Productos Bio-Logicos, Argentina) according to the manufacturer’s instructions. The intensity of the bands was quantified using optical densitometry (Scion Image Release Beta 4.0.2, NIH, United States). After densitometry of immunoblots, values of the RD group were normalized to 100%, and both SRD and SRD + Ca were expressed relative to this (Supplementary material). The protein levels were normalized to those of β-actin.

### Statistical analysis

Results are represented as mean ± SEM values. Comparisons among dietary groups utilized a transverse approach while variance homogeneity was checked via Levene’s test and data normality through Shapiro-Wilk testing methods. One-way ANOVA analyzed statistical differences among groups—RD, SRD, and SRD + Ca—followed by Newman-Keuls *post hoc* testing where significance was established at *p* < 0.05 level using SPSS software version 17.0 for Windows (SPSS Inc., Chicago, Illinois, United States).

## Results

### NAS score and hepatic enzyme activities related to lipid metabolism

Table 3 shows that SRD-fed rats developed a significant increase in NAS score (Score: 3) compared to the RD group (*P* < 0.05), indicating the presence of liver steatosis and associated histopathological alterations. Oral administration of cannabis oil significantly improved the NAS score in the SRD + Ca group, restoring values close to those observed in RD-fed rats (*P* < 0.05).

**TABLE 3:** Histologic scoring system for activity grade of non-alcoholic fatty liver disease (NAS) in liver sections in female rats fed a reference diet (RD), sucrose-rich diet (SRD) or SRD with cannabis oil (SRD + Ca).

Histologic scoring system	RD		SRD		SRD + Ca	
	%	Scoring	%	Scoring	%	Scoring
Steatosis	< 5%	0	23%	1	<5	0
Hepatocelullar ballooning	None	0	Few balloned cells	1	Few balloned cells	1
Lobular inflammation	None	0	< 2foci/20x field	1	None	0
NAFLD activity score (NAS)		0		3		1

Values are expressed as mean ± SEM, *n* = 6.

Consistent with these histological findings, SRD feeding induced a marked increase in the activities of key enzymes involved in *de novo* lipogenesis, including ACC, FAS, G-6-PDH, and ME, in liver tissue compared to the RD group (Table 4). Cannabis oil administration attenuated these alterations, as evidenced by a significant reduction in ACC and ME activities (*P* < 0.05), which returned to levels comparable to those of RD-fed rats. Although FAS and G-6-PDH activities were also reduced in the SRD + Ca group, their values remained higher than those observed in the RD group.

**TABLE 4:** Enzymes involved in hepatic steatosis in female rats fed a reference diet (RD), sucrose-rich diet (SRD) or SRD with cannabis oil (SRD + Ca).

Enzymes hepatic steatosis	RD	SRD	SRD + Ca
ACC (mU/mg protein)	52.07 ± 3.26	75.98 ± 5.38*	58.58 ± 3.39
FAS (mU/mg protein)	6.00 ± 0.51	10.44 ± 0.55*	8.02 ± 0.74^#^
G-6-p DH (mU/mg protein)	30.12 ± 3.57	81.28 ± 9.45*	60.37 ± 4.95^#^
EM (mU/mg protein)	27.51 ± 2.69	44.91 ± 3.08*	31.50 ± 1.68
CPT-1 (mU/mg protein)	1.15 ± 0.11	0.71 ± 0.05*	1.09 ± 0.06
CPT-2 (mU/mg protein)	16.57 ± 0.87	14.72 ± 0.50	15.97 ± 0.68
Total-CPT (mU/mg protein)	19.86 ± 0.87	16.11 ± 0.42*	18.93 ± 0.41

Values are expressed as mean ± SEM. *n* = 6. Statistical differences were evaluated by one-way ANOVA followed by the Newman–Keuls *post-hoc* test (*P < 0.05 and ^#^*P* < 0.05 vs. SRD and RD).

In parallel, the activities of CPT enzymes, which are essential for mitochondrial fatty acid oxidation, were significantly decreased in the liver of SRD-fed rats, as shown by reduced CPT-1 and total-CPT activities compared to the RD group (< 0.05) (Table 4). Cannabis oil treatment significantly increased CPT-1 and total-CPT activities in the SRD + Ca group, restoring them to values similar to those observed in RD-fed rats, while CPT-2 activity remained unchanged across all experimental groups.

### Liver lipid peroxidation and oxidative stress biomarkers

**FIGURE 1:**
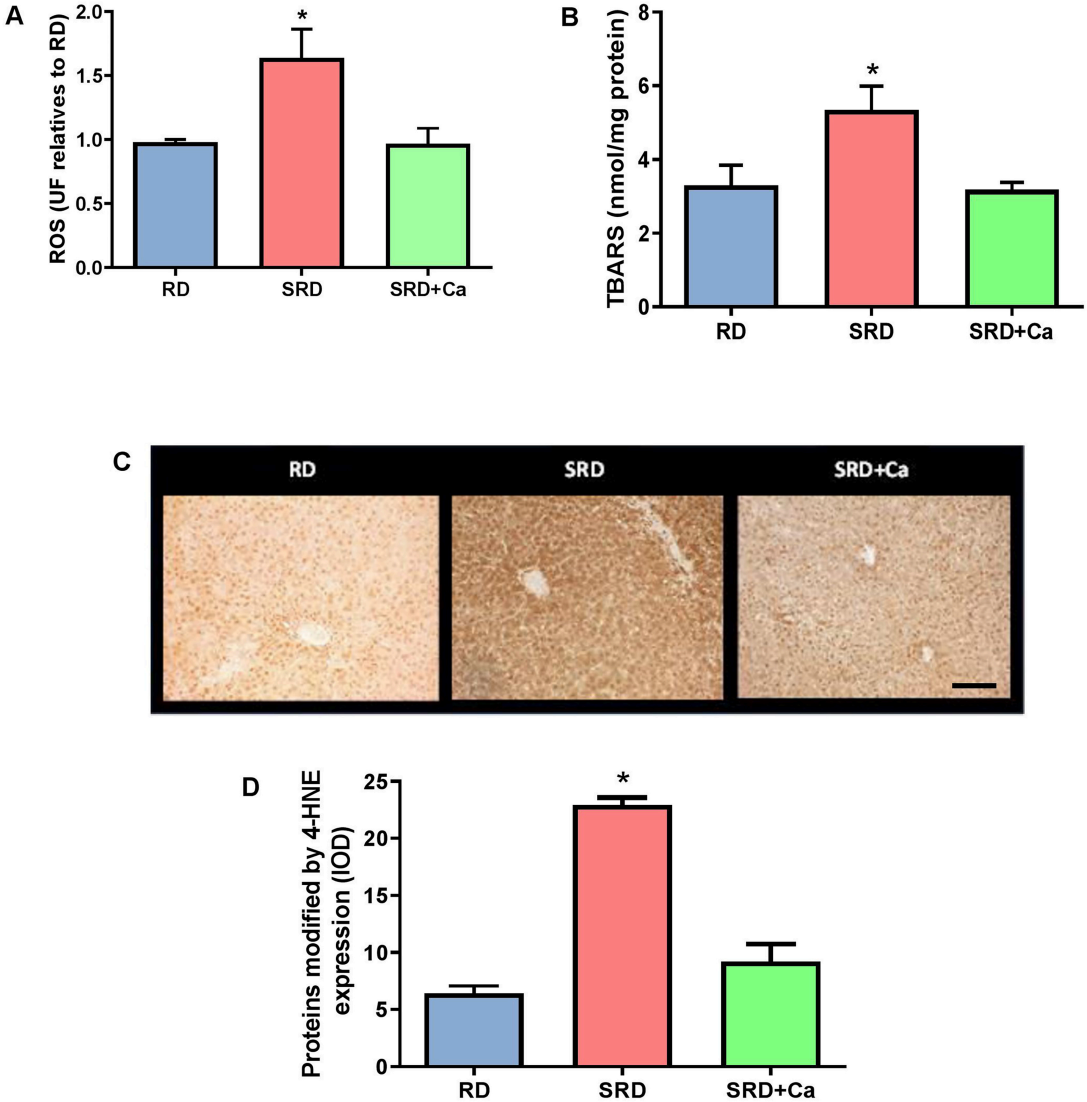
Liver lipid peroxidation and oxidative damage markers in female rats fed a reference diet (RD), sucrose-rich diet (SRD) or SRD with cannabis oil (SRD + Ca). **(A)** Liver reactive oxygen species (ROS) levels. **(B)** Liver thiobarbituric acid reactive substances (TBARS). **(C)** Representative photomicrographs of 4-HNE expression in liver sections of rats. An increase of hepatocytes with nuclear and cytoplasmic positive markers is observed in the SRD group. Scale bar 50 μm. **(D)** Quantitative immunohistochemical analysis of 4-HNE–modified proteins expressed as integrated optical density (IOD). Data are expressed as mean ± SEM (*n* = 6). Statistical differences were evaluated by one-way ANOVA followed by the Newman–Keuls *post-hoc* test (*P < 0.05).

Liver ROS levels were significantly increased in the SRD group compared to the RD group (*P* < 0.05; Figure 1A), indicating enhanced oxidative stress. Oral administration of cannabis oil to SRD-fed rats resulted in a marked reduction in ROS levels (*P* < 0.05), restoring values close to those observed in the RD group. In parallel, liver lipid peroxidation, assessed by TBARS content, was significantly elevated in the SRD group relative to RD-fed rats (*P* < 0.05) (Figure 1B). This increase was significantly attenuated in the SRD + Ca group, with TBARS levels returning to values comparable to those of the RD group (*P* < 0.05).

Consistent with these biochemical findings, immunohistochemical analysis revealed a marked increase in 4-HNE–modified proteins in the liver of SRD-fed rats compared to the RD group (*P* < 0.05) (Figures 1C,D). Representative photomicrographs (Figure 1C) showed an increased number of hepatocytes displaying positive 4-HNE immunostaining, with signal localized in both cytoplasmic and nuclear compartments in the SRD group.

Quantitative image analysis confirmed these observations, demonstrating a significant increase in integrated optical density (IOD) of 4-HNE immunoreactivity in SRD-fed rats relative to RD-fed animals (*P* < 0.05) (Figure 1D). Cannabis oil administration significantly reduced 4-HNE immunostaining in the SRD + Ca group, as evidenced by both representative images and IOD quantification (*P* < 0.05). However, 4-HNE levels in the SRD + Ca group remained higher than those observed in the RD group.

**FIGURE 2:**
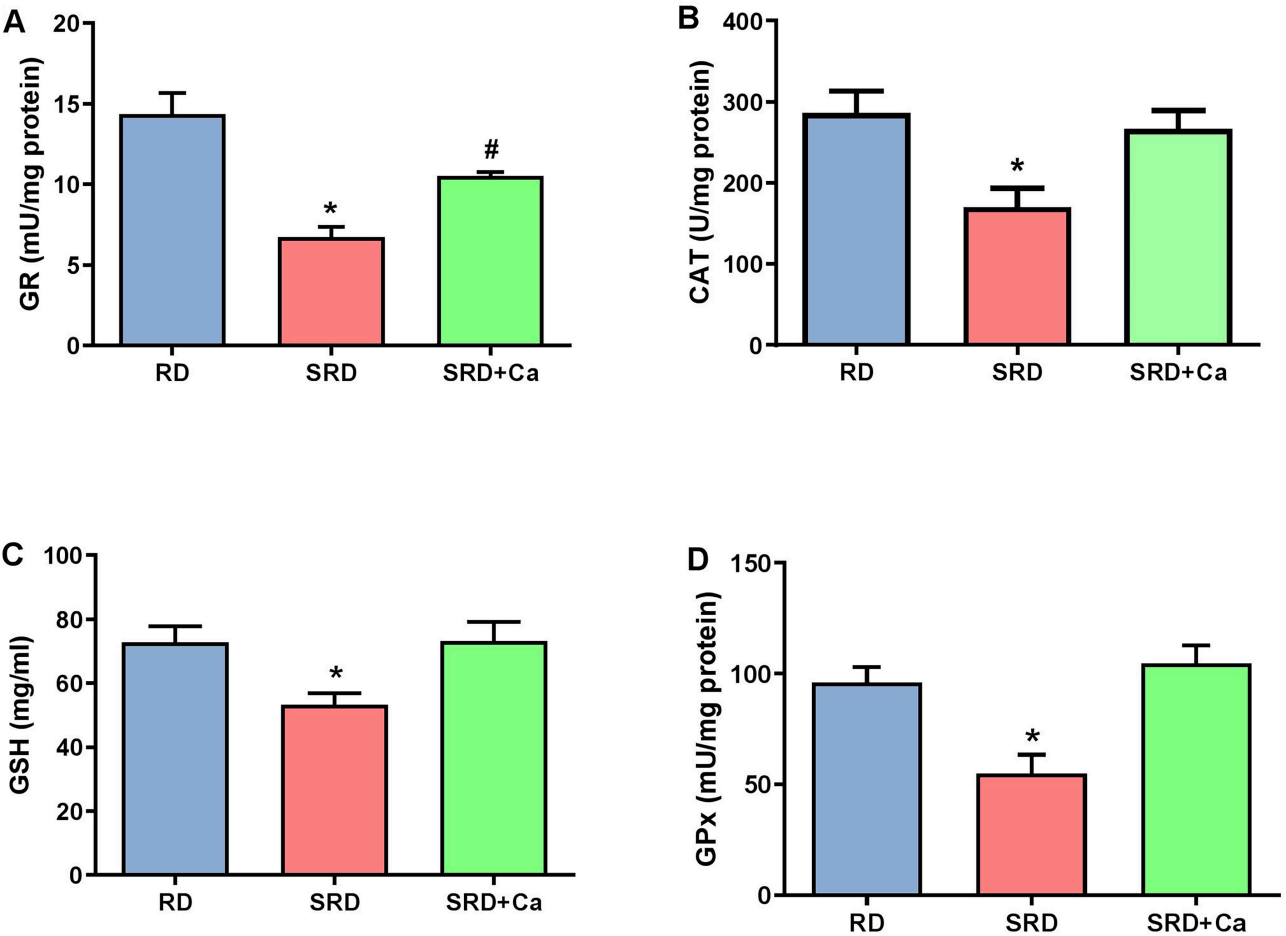
Liver antioxidant defenses and redox status in female rats fed a reference diet (RD), sucrose-rich diet (SRD) or SRD with cannabis oil (SRD + Ca). **(A)** Liver glutathione (GSH) content. **(B)** Catalase (CAT) activity. **(C)** Glutathione peroxidase (GPx) activity and **(D)** Glutathione reductase (GR) activity. Data are expressed as mean ± SEM (*n* = 6). Statistical differences were evaluated by one-way ANOVA followed by the Newman–Keuls *post-hoc* test (*P < 0.05 and ^#^*P* < 0.05 vs. SRD and RD).

Alterations in oxidative stress were further reflected by changes in the antioxidant defense system. Liver GSH content was significantly reduced in the SRD group compared to the RD group (*P* < 0.05) (Figure 2A), whereas cannabis oil administration restored GSH levels to values similar to those observed in RD-fed rats. In addition, the activities of antioxidant enzymes CAT, GPx, and GR were significantly decreased in the SRD group (*P* < 0.05) (Figures 2B–D). Cannabis oil treatment partially increased CAT activity, although values remained lower than those of the RD group, while GPx and GR activities were significantly enhanced in the SRD + Ca group (*P* < 0.05), reaching levels comparable to those observed in RD-fed rats.

### Liver NFκB p65 and NrF2

**FIGURE 3:**
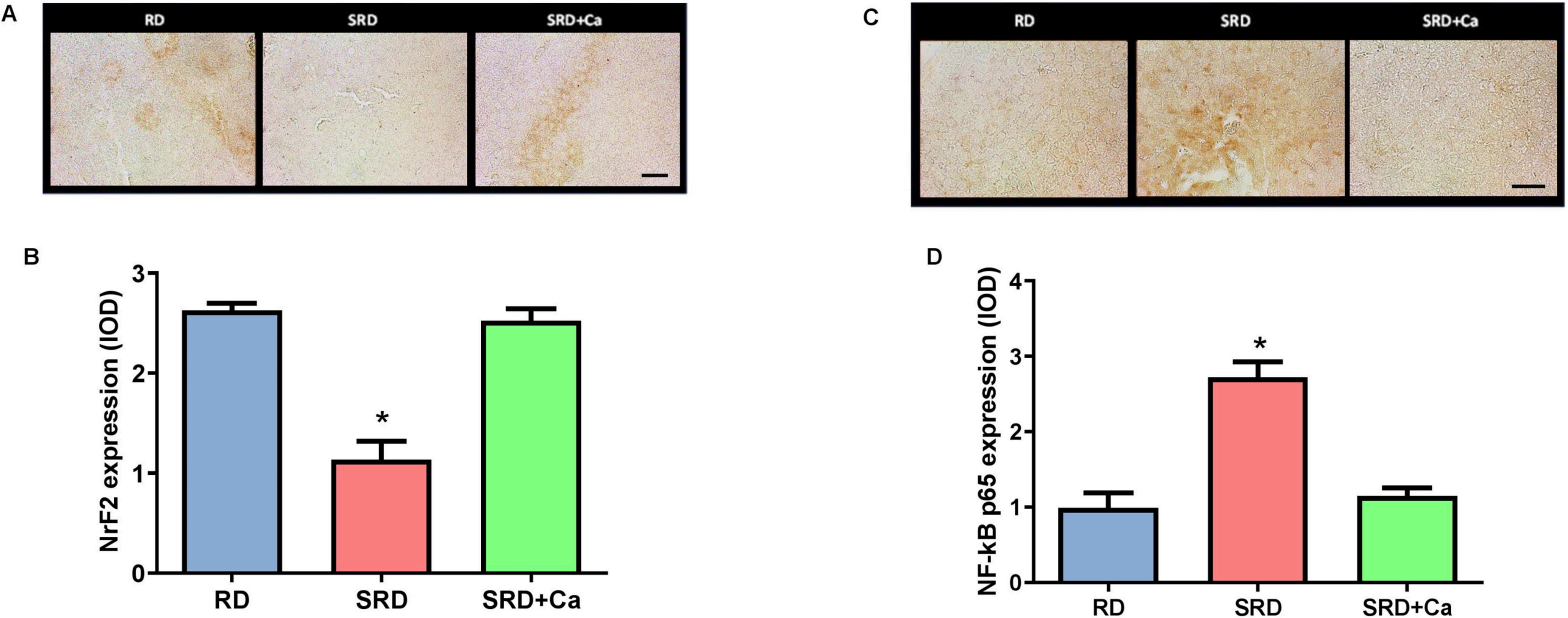
Liver NrF2 and NFκB p65 expression in female rats fed a reference diet (RD), sucrose-rich diet (SRD) or SRD with cannabis oil (SRD + Ca). **(A)** Representative photomicrographs of immunocytochemical staining NrF2 in the liver sections of rats. Decreased levels of nuclear and cytoplasmic positive markers are observed in the SRD group. Scale bar 50 μm. **(B)** Quantitative immunohistochemical analysis of liver NrF2 expression expressed as integrated optical density (IOD). **(C)** Representative photomicrographs of immunocytochemical staining NFκB p65 in the liver sections of rats. Increased levels of nuclear and cytoplasmic positive markers are observed in the SRD group. Scale bar 50 μm. **(D)** Quantitative immunohistochemical analysis of NFκB p65 expression in the liver. Data are expressed as mean ± SEM (*n* = 6). Statistical differences were evaluated by one-way ANOVA followed by the Newman–Keuls *post hoc* test (*P < 0.05).

Immunohistochemical analysis revealed significant alterations in the expression of NrF2 and NF-κB p65 in the liver of rats fed an SRD (Figure 3). Representative photomicrographs and quantitative analysis showed that NrF2 expression was significantly reduced in the SRD group compared to the RD group (*P* < 0.05) (Figures 3A,B). NrF2 immunostaining was detected in both nuclear and cytoplasmic compartments. Cannabis oil administration significantly increased NrF2 protein expression in the SRD + Ca group relative to SRD-fed rats (*P* < 0.05), although NrF2 levels remained lower than those observed in the RD group.

In contrast, NF-κB p65 expression was markedly increased in the liver of SRD-fed rats compared to RD-fed animals (*P* < 0.05) (Figures 3C,D). Representative images showed enhanced NF-κB p65 immunoreactivity in both nuclear and cytoplasmic regions in the SRD group. Quantitative analysis confirmed a significant elevation in NF-κB p65 expression relative to the RD group (*P* < 0.05). Treatment with cannabis oil significantly reduced NF-κB p65 protein levels in the SRD + Ca group compared to the SRD group (*P* < 0.05), restoring values close to those observed in RD-fed rats.

### Liver fibrosis

**FIGURE 4:**
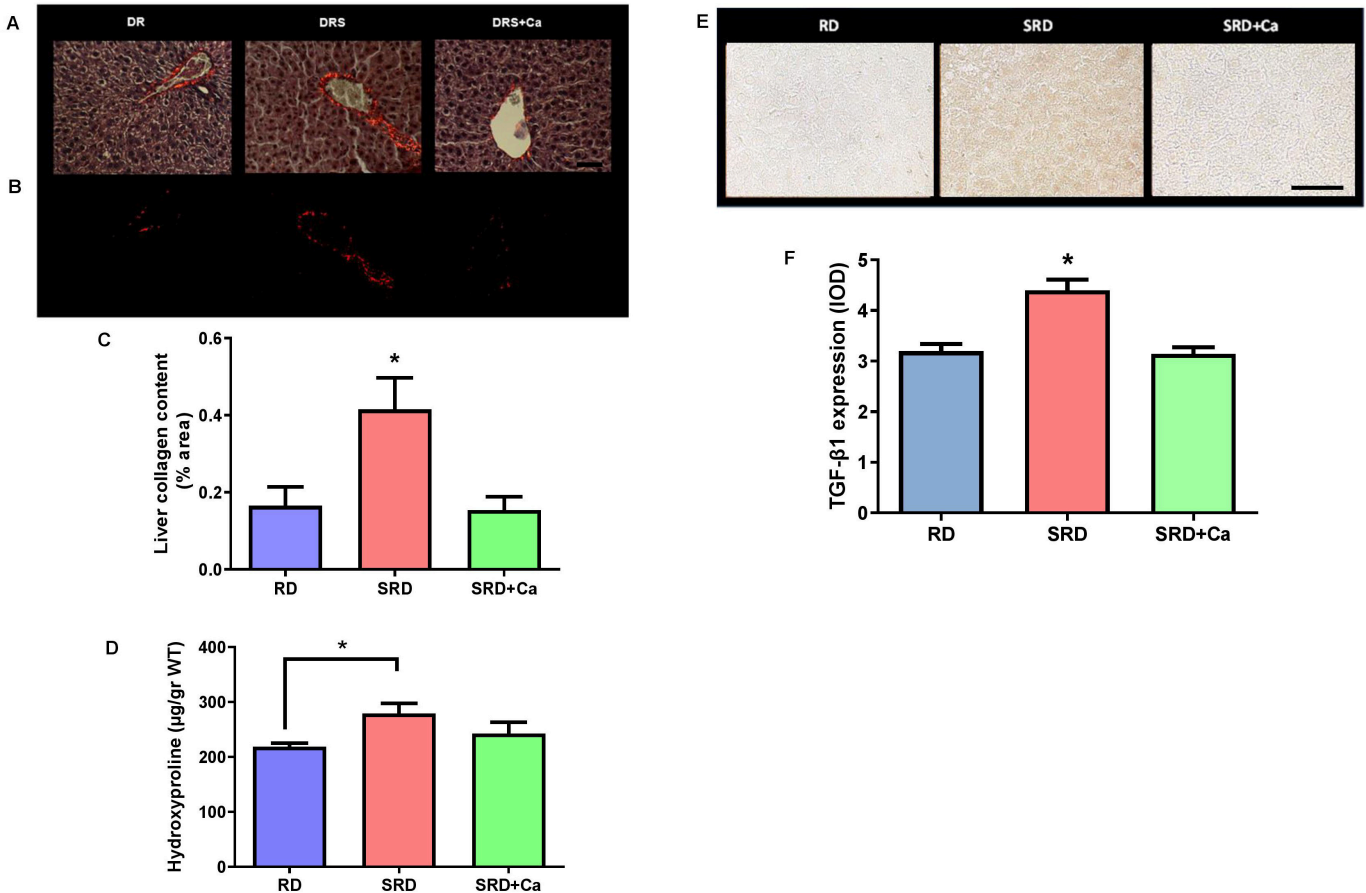
Liver collagen, hydroxyproline content and TGF-β1 expression in female rats fed a reference diet (RD), sucrose-rich diet (SRD) or SRD with cannabis oil (SRD + Ca). **(A)** Picrosirius-stained histological sections of the liver. Abnormal collagen deposition is observed around the hepatocytes and blood vessels in the SRD group. Scale bar 50 μm. **(B)** Polarized light. Scale bar 50 μm. **(C)** Quantification of the collagen-positive area in the liver sections by polarized light microscopy. **(D)** Hydroxyproline content of the liver. **(E)** Representative photomicrographs of immunocytochemical staining TGF- β1 in the liver sections of rats. **(F)** Quantitative immunohistochemical analysis of expression of TGF-β1 in liver. Data are expressed as mean ± SEM (*n* = 6). Statistical differences were evaluated by one-way ANOVA followed by the Newman–Keuls *post-hoc* test (*P < 0.05).

Histological examination revealed early fibrotic changes in the liver of SRD-fed rats, characterized by increased collagen fiber deposition within vascular walls and interstitial areas compared to the RD group (Figure 4A). These observations were quantitatively confirmed using picrosirius red staining under polarized light, which showed a significantly higher percentage of collagen-positive areas in the SRD group relative to RD-fed rats (*P* < 0.05) (Figures 4B,C).

Consistent with these histological findings, liver hydroxyproline content—a biochemical marker of collagen accumulation—was significantly increased in the SRD group compared to the RD group (*P* < 0.05) (Figure 4D). Cannabis oil administration significantly attenuated collagen deposition, as evidenced by a reduction in both collagen-positive area and hydroxyproline levels in the SRD + Ca group (*P* < 0.05), restoring values close to those observed in RD-fed rats.

In parallel, immunohistochemical analysis of TGF-β1 expression revealed a significant increase in TGF-β1 protein levels in the liver of SRD-fed rats compared to the RD group (*P* < 0.05) (Figures 4E,F). Representative photomicrographs showed enhanced TGF-β1 immunostaining predominantly localized around blood vessels. Quantitative analysis confirmed these observations, demonstrating a significant reduction in TGF-β1 expression in the SRD + Ca group compared to the SRD group (*P* < 0.05), with levels comparable to those detected in RD-fed rats.

### Endocannabinoid system

**FIGURE 5:**
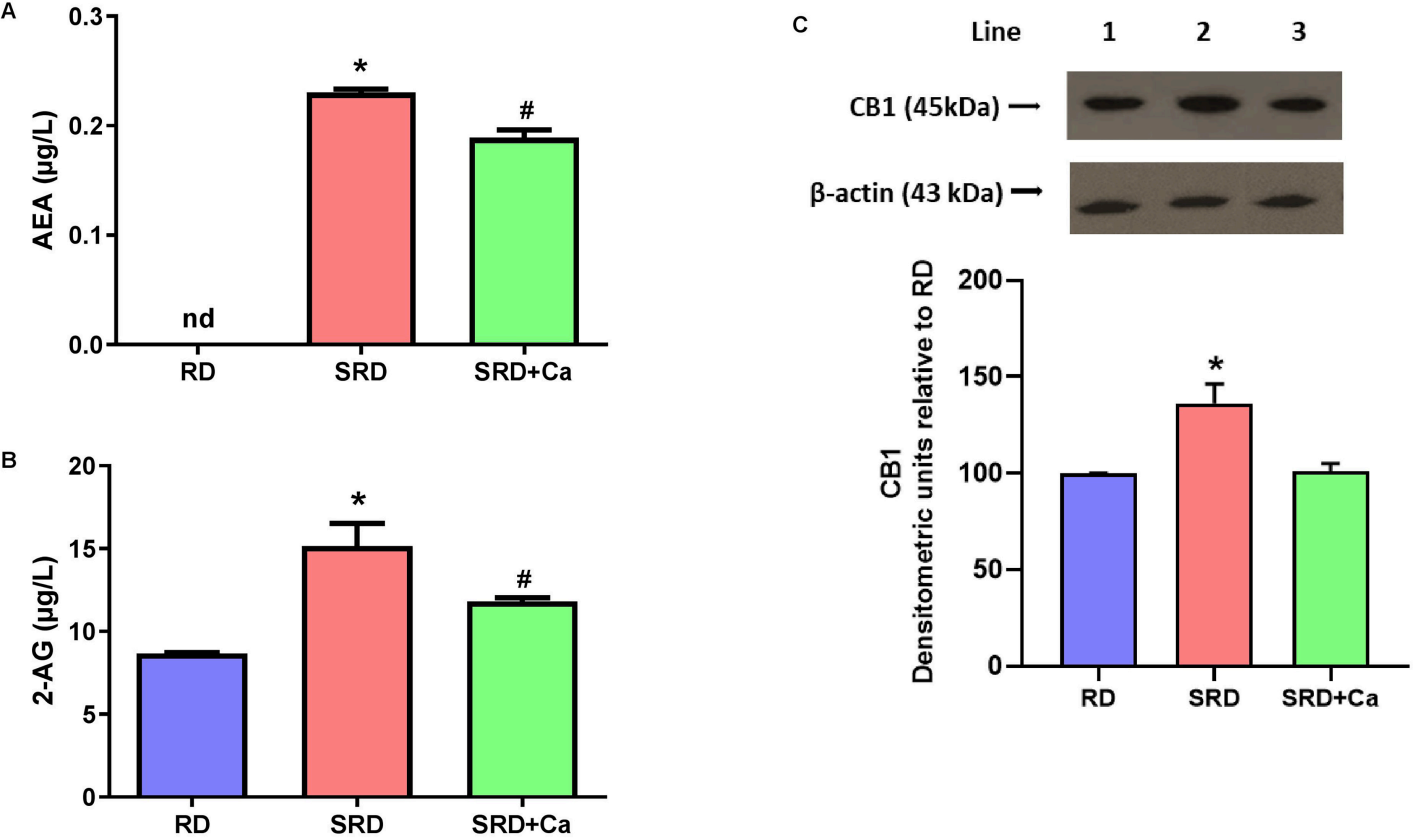
Endocannabinoids serum levels and liver CB1 protein mass in female rats fed a reference diet (RD), sucrose-rich diet (SRD) or SRD with cannabis oil (SRD + Ca). **(A)** Serum anandamide (AEA) levels. **(B)** Serum 2-arachidonoylglycerol (2-AG) levels. **(C)** Liver protein mass levels of CB1. Each gel contained an equal number of samples from rats fed a RD, SRD and SRD + Ca. Upper. Representatives immunoblot of liver CB1. Bottom. Densitometric immunoblot analysis of the CB1 protein mass levels. Data are expressed as mean ± SEM (*n* = 6). Statistical differences were evaluated by one-way ANOVA followed by the Newman–Keuls *post-hoc* test (*P < 0.05 and ^#^*P* < 0.05 vs. SRD and RD).

Alterations in the endocannabinoid system were observed in SRD-fed rats, as evidenced by significantly increased serum levels of the endocannabinoids AEA and 2-AG compared to the RD group (Figures 5A,B). Cannabis oil administration significantly reduced the circulating concentrations of both endocannabinoids in the SRD + Ca group (*P* < 0.05); however, their levels remained higher than those observed in RD-fed rats.

Consistent with these systemic changes, Western blot analysis revealed a significant increase in CB1 protein expression in the liver of SRD-fed rats compared to the RD group (*P* < 0.05) (Figure 5C). Both qualitative immunoblot visualization and densitometric quantification confirmed this increase. In contrast, cannabis oil treatment significantly reduced liver CB1 protein levels in the SRD + Ca group relative to the SRD group (*P* < 0.05), restoring values to levels comparable to those observed in RD-fed rats.

## Discussion

MASLD is strongly associated with disruptions in the endocannabinoid system (ECS) in both human and animal models. Stimulation of the CB1 receptor has been shown to increase the expression of liver lipogenic genes while concurrently decreasing CPT-1 function, a key enzyme involved in fatty acid oxidation. These changes can promote liver oxidative stress, lipid peroxidation, inflammation, and fibrosis. In our study, rats fed a sucrose-rich diet (SRD) exhibited increased serum endocannabinoid levels, a significant rise in enzymes related to *de novo* fatty acid synthesis and NAS score, and a reduction in enzymes responsible for mitochondrial fatty acid oxidation. Collectively, these findings suggest that sucrose intake disrupts lipid metabolism by favoring synthesis over oxidation, thereby facilitating triglyceride accumulation in hepatocytes and the development of liver steatosis. In addition, SRD-fed rats showed a significant increase in lipid peroxidation markers (TBARS and 4-HNE) and ROS levels, alongside reduced GSH content and decreased activities of antioxidant enzymes (CAT, GPx, and GR), consistent with an enhanced oxidative stress milieu. The molecular pathways involved in MASLD progression include transcription factors such as NrF2 and NF-κB; NrF2 is known to antagonize NF-κB signaling through multiple mechanisms. In line with this, SRD feeding was associated with reduced liver NrF2 expression and increased NF-κB p65 levels. Finally, SRD-fed rats also displayed early profibrotic changes, including increased interstitial collagen deposition, hydroxyproline content, and TGF-β1 expression in liver tissue, together with elevated CB1 expression. Notably, a positive correlation between circulating 2-AG and liver fat content has been reported in women with NAFLD, supporting the concept that systemic ECS tone may relate to liver lipid accumulation and potentially contribute to enhanced *de novo* lipogenesis.

CB1, a key component of the ECS, plays a central role in lipid metabolism regulation and is involved in the pathogenesis of liver steatosis. CB1 activation promotes fat accumulation by increasing liver lipogenesis and reducing fatty acid oxidation. Previous studies show that CB1 receptor activation enhances lipogenic transcription factors and their downstream enzymes in the liver, which is a major site of *de novo* fatty acid synthesis. Accordingly, liver CB1 blockade or genetic disruption has been associated with reduced lipogenesis and improvements in hypercholesterolemia, hypertriglyceridemia, and liver injury. Beyond lipid metabolism, CB1 activation is also linked to oxidative stress and fibrosis development. By increasing ROS production and impairing antioxidant defenses, CB1 activation can aggravate oxidative damage; additionally, CB1-mediated pathways can promote fibrogenesis by stimulating hepatic stellate cell proliferation and collagen deposition, whereas CB1 antagonism has been associated with antifibrotic effects in experimental models.

Against this pathophysiological background, the growing medical acceptance of cannabinoids and the expanding understanding of ECS biology have stimulated research into cannabis-based interventions. THC and CBD remain the best-characterized phytocannabinoids, yet full-spectrum preparations contain a broader array of cannabinoids and other compounds (e.g., terpenes and flavonoids) that may interact additively or synergistically (“entourage”-like effects). Phytocannabinoids may act in concert with terpenes, flavonoids, and other minor compounds to modulate pharmacological responses through additive or synergistic interactions, potentially enhancing efficacy while improving tolerability (6, 7). Several terpenes commonly found in Cannabis sativa—such as myrcene, limonene, and β-caryophyllene—have been reported to exert antioxidant, anti-inflammatory, and metabolic effects, as well as to interact with signaling pathways relevant to liver homeostasis. Importantly, because the intervention tested here is a full-spectrum, plant-derived oil, the observed biological responses should be interpreted as the integrated effect of the preparation as a whole rather than ascribable to a single compound. This design choice aligns with the real-world composition of many medicinal cannabis products and is therefore relevant from a translational perspective.

In this study, oral administration of full-spectrum cannabis oil (CBD:THC 2:1) effectively prevented SRD-induced liver steatosis and improved the lipogenic/oxidative lipid pathway, together with a normalization trend in circulating endocannabinoids and reduced liver CB1 expression. These findings are consistent with experimental evidence indicating hepatoprotective actions of cannabinoids in NAFLD/MASLD settings, including antisteatotic effects reported for CBD in murine models. Of note, studies using cannabis extracts with different cannabinoid enrichments have reported divergent outcomes, highlighting that cannabinoid ratio and extract composition may critically shape metabolic responses. Therefore, our data add evidence that a defined full-spectrum preparation with a CBD:THC ratio of 2:1 is associated with improved MASLD-related outcomes in this dietary model.

Cannabinoids have been described to exert antioxidant actions through both direct free radical scavenging and indirect modulation of redox balance, including regulation of GSH levels, activation of antioxidant enzymes, and inhibition of pro-oxidant pathways. In our study, cannabis oil administration reduced lipid peroxidation (TBARS and 4-HNE) and ROS levels, and improved both enzymatic (CAT, GR, GPx) and non-enzymatic (GSH) components of the liver antioxidant defense system. Moreover, treatment increased NrF2 expression while decreasing NF-κB p65 levels, consistent with a shift toward an antioxidant/anti-inflammatory transcriptional environment. Prior work has shown that CBD can reduce oxidative damage markers and inflammatory signaling across different liver injury paradigms, and can engage NrF2-related antioxidant pathways, supporting the plausibility of the pattern observed here. While our study does not establish causal molecular mechanisms, the coordinated improvement in oxidative stress biomarkers and NrF2/NF-κB signaling is consistent with an overall attenuation of SRD-induced oxidative and inflammatory stress.

The ECS also plays a role in liver fibrogenesis. In cirrhosis, CB1 receptors are upregulated and expressed in fibrogenic liver cells, and CB1 inhibition has been linked to reduced fibrosis and lower TGF-β signaling. In our model, cannabis oil administration improved multiple fibrosis-related endpoints, including reduced collagen deposition, lower hydroxyproline content, and decreased TGF-β1 expression. These results align with experimental observations that cannabinoids—particularly CBD—may modulate immune infiltration, fibroblast migration, and collagen gene programs in fibrotic settings. Taken together, the present findings support the concept that modulation of ECS tone and CB1 expression is associated with improvement across steatosis–oxidative stress–fibrosis axes in diet-induced MASLD.

A key strength of the present work is the exclusive use of female rats, addressing the persistent male bias in preclinical MASLD research. MASLD is increasingly recognized as a sex-dimorphic condition: during reproductive age, females generally show lower prevalence and/or severity than males, whereas risk of advanced fibrosis can rise after menopause, suggesting a protective role for estrogens and sex-dependent metabolic and immune regulation (33–35).

Mechanistically, estrogens have been reported to promote fatty acid oxidation, suppress *de novo* lipogenesis, and modulate mitochondrial function, oxidative stress responses, and inflammatory signaling—pathways directly implicated in MASLD progression. In parallel, accumulating evidence indicates bidirectional crosstalk between sex hormones and the ECS, with sex differences described in cannabinoid pharmacology and ECS component expression/function; females can show distinct sensitivity profiles to cannabinoids and ECS signaling, potentially influenced by ovarian hormones (17, 18, 36).

Our findings in females—namely, SRD-induced ECS overactivation (higher AEA/2-AG and increased liver CB1) together with improvements in the lipogenesis/β-oxidation balance, oxidative stress biomarkers, and fibrosis-related endpoints following cannabis oil administration—support the relevance of evaluating ECS-targeted nutritional interventions in a female biological context. The female-specific model strengthens the novelty of the work by expanding current evidence beyond male-centered studies and by providing a basis for future investigations testing whether hormonal status (e.g., estrous cycle phase, ovariectomy, or menopause-like models) modifies ECS regulation and responses to full-spectrum cannabinoid preparations in MASLD (37, 38).

One limitation of this study is the use of a full-spectrum cannabis oil, which, despite being chemically characterized and translationally relevant, does not allow attribution of the observed effects to individual cannabinoids or other bioactive compounds. Future studies using isolated compounds or targeted cannabinoid or terpene formulations will be required to establish causal relationships and define specific mechanisms of action. In addition, the experimental design relied on a single dose and a short-term intervention focused on early stages of diet-induced MASLD. While appropriate to evaluate preventive effects, this approach precludes assessment of dose–response relationships, therapeutic windows, and long-term outcomes, particularly regarding fibrosis progression and metabolic recovery. Finally, mechanistic analyses were necessarily selective. Although key markers of fibrosis, oxidative stress, and endocannabinoid system alterations were evaluated, a more comprehensive characterization—including hepatic stellate cell activation, upstream sources of oxidative stress, additional ECS components, and endocannabinoid-metabolizing enzymes—would further strengthen mechanistic insight.

## Conclusion

This study identified new mechanisms through which cannabis oil, specifically with a CBD:THC ratio of 2:1, alleviates liver steatosis caused by SRD in female rats. The study revealed a reduction in the activity of lipogenic enzymes alongside an increase in CPT-1 enzyme activity, which is essential for mitochondrial fatty acid oxidation. Furthermore, cannabis oil administration was associated with reduced lipid peroxidation and oxidative stress, together with a coordinated modulation of redox-sensitive transcription factors, characterized by increased NrF2 and decreased NF-κB p65 expression. While NF-κB is not a direct mediator of lipid oxidation, its downregulation may reflect attenuation of pro-inflammatory signaling that contributes to oxidative and metabolic imbalance. Additionally, we demonstrated that cannabis oil with a CBD:THC 2:1 ratio effectively reduces liver fibrosis. Importantly, this study established for the first time a connection between the ECS and liver lipid metabolism, oxidative stress, and fibrosis in SRD-fed female rats over a duration of 3 weeks. Our results indicate that cannabis oil with this particular CBD:THC ratio may serve as a natural nutraceutical to help prevent metabolic disorders linked to hepatic steatosis, oxidative stress, liver fibrosis, and MASLD. While it remains possible that other components of cannabis oil—such as terpenes, flavonoids, and alkaloids—may have contributed to these positive effects, the findings are encouraging. However, additional research is necessary before considering full-spectrum cannabis oil as an adjunct nutraceutical for addressing metabolic disorders in humans.

## Data Availability

The original contributions presented in the study are included in the article/Supplementary material, further inquiries can be directed to the corresponding authors.
