# Supplementary material for: Cannabis oil modulates liver alterations and endocannabinoid system changes in a female rat model of diet-induced MASLD
Source: Front Nutr. 2026 Mar 10;13:1770150. doi: 10.3389/fnut.2026.1770150 (PMC13008911; doi:10.3389/fnut.2026.1770150)

CB1 Western Blott

	Sample	Sample-blank	b-actin	b-actin-blanck	Result	% RD
**Blanck**	180,11	5,74	159,13	15,34	0,3742	87,24
**SRD+CA 246**	185,85		174,47			
**Blanck**	180,11	8,23	159,13	19,14	0,4300	100,25
**SRD+CA 247**	188,34		178,27			
**Blanck**	180,11	4,44	159,13	8,7	0,5103	118,99
**SRD 238**	184,55		167,83			
**Blanck**	180,11	11,25	159,13	26,23	0,4289	100,00
**RD 236**	191,36		185,36			
**Blanck**	180,11	10,47	159,13	19,13	0,5473	127,61
**SRD 239**	190,58		178,26			
**Blanck**	180,11	9,5	159,13	21,01	0,4522	105,42
**SRD 249**	189,61		180,14			
**Blanck**	180,11	8,72	159,13	19,09	0,4568	106,50
**SRD+CA 252**	188,83		178,22			

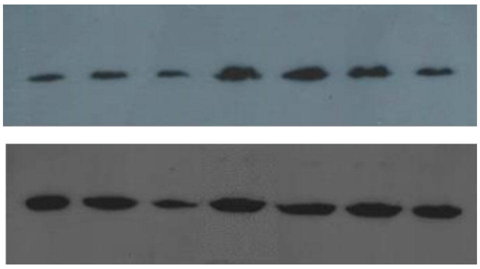

	Sample	Sample-blank	b-actin	b-actin-blanck	Result	% RD
**Blanck**	136,81	28,46	156,39	22,63	1,2576	91,83
**SRD+CA 253**	165,27		179,02			
**Blanck**	136,81	31,86	156,39	20,15	1,5811	115,46
**SRD+CA 254**	168,67		176,54			
**Blanck**	136,81	32,1	156,39	21,15	1,5177	110,83
**SRD 250**	168,91		177,54			
**Blanck**	136,81	36,25	156,39	26,47	1,3695	100,00
**RD 242**	173,06		182,86			
**Blanck**	136,81	39,11	156,39	24,98	1,5657	114,33
**SRD 249**	175,92		181,37			
**Blanck**	136,81	29,06	156,39	21,78	1,3343	97,43
**SRD 238**	165,87		178,17			
**Blanck**	136,81	27,8	156,39	20,84	1,3340	97,41
**SRD+CA 248**	164,61		177,23			

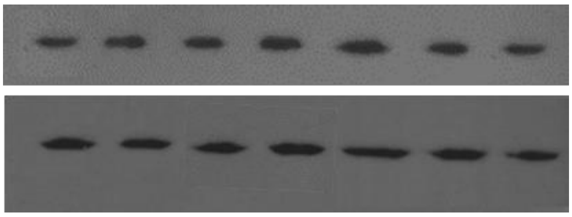

	Sample	Sample-blank	b-actin	b-actin-blanck	Result	% RD
**Blanck**	111,84	40,7	117,67	18,15	2,2424	166,92
**DRS 240**	152,54		135,82			
**Blanck**	143,79	13,49	117,67	5,99	2,2521	167,64
**DRS 251**	157,28		123,66			
**Blanck**	143,79	8,85	117,67	8	1,1063	82,35
**DRS+CA 254**	152,64		125,67			
**Blanck**	143,79	18,27	114,69	13,6	1,3434	100,00
**DR 237**	162,06		128,29			
**Blanck**	143,79	17,44	117,67	10,65	1,6376	121,90
**DRS 250**	161,23		128,32			
**Blanck**	143,79	10,56				
**DRS+CA 247**	154,35					
**Blanck**	140,9	5,35	117,67	3,99	1,3409	99,81
**DRS+CA 252**	146,25		121,66			

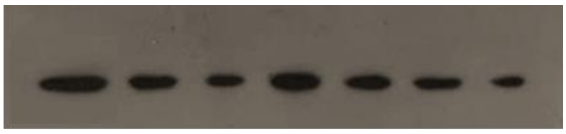

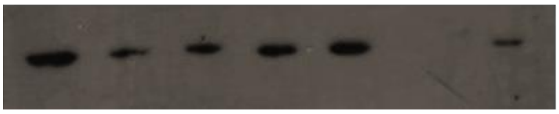

Supplement: Supplementary file 1 [file Data_Sheet_1.pdf]
