# Supplementary figures and images for: Cannabis oil modulates liver alterations and endocannabinoid system changes in a female rat model of diet-induced MASLD
Source: Front Nutr. 2026 Mar 10;13:1770150. doi: 10.3389/fnut.2026.1770150 (PMC13008911; doi:10.3389/fnut.2026.1770150)

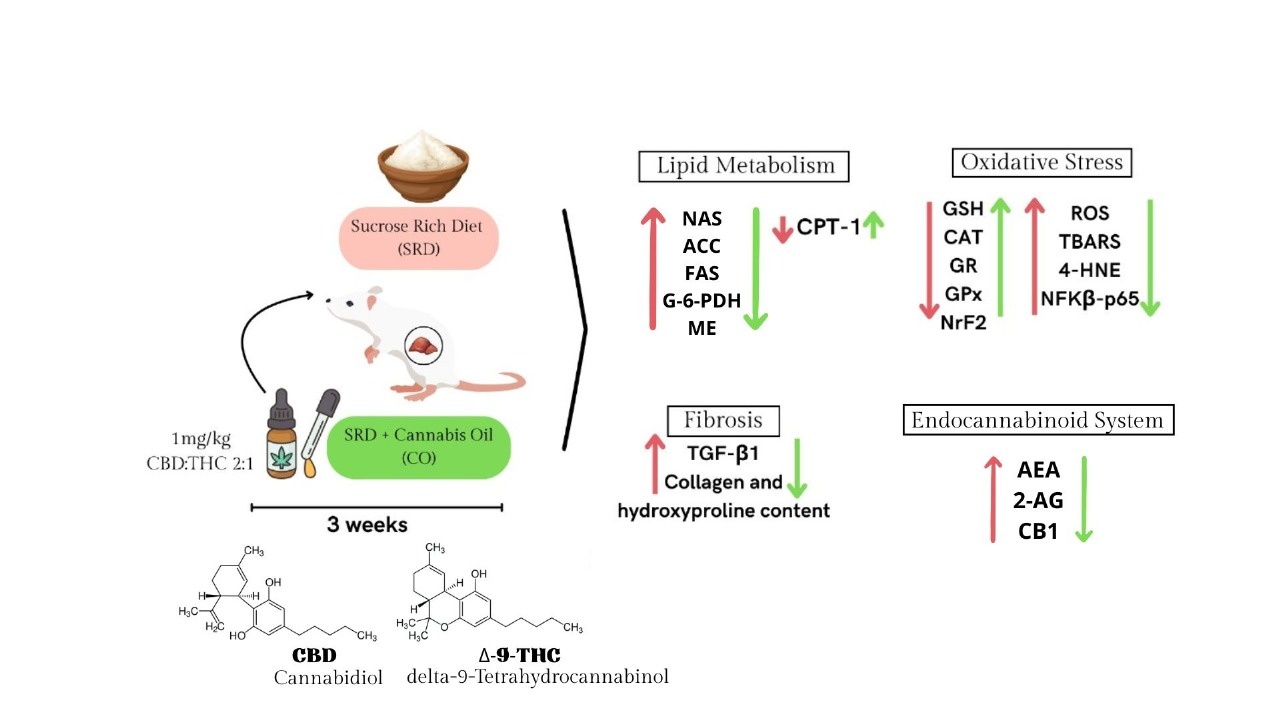

Supplement: Supplementary file 2 [file Image_1.JPEG]
